# Spectral overlap of far-red light with chlorophyll fluorescence causes artifacts in PAM-based *Φ*_PSII_ determination

**DOI:** 10.3389/fpls.2026.1703422

**Published:** 2026-01-28

**Authors:** Ji Up Park, Youbin Zheng, Jongyun Kim

**Affiliations:** 1 Department of Plant Biotechnology, Korea University, Seoul, Republic of Korea; 2 School of Environmental Science, University of Guelph, Guelph, ON, Canada

**Keywords:** light-emitting diode, interference, light geometry, measurement artifact, quantum yield

## Abstract

Far-red light (FR, 700–800 nm) can enhance photosynthesis by stimulating photosystem I (PSI). However, during chlorophyll fluorescence (CF) measurements using pulse-amplitude modulation (PAM) fluorometry, unusually high quantum yields of photosystem II (*Φ*_PSII_) have been observed under high FR light intensities, raising concerns about measurement artifacts. To test this, we constructed light response curves for sweet basil (*Ocimum basilicum* L.) grown under light-emitting diode (LED) light (R:G:B = 44%:18%:38%) with varying photosynthetic photon flux densities (PPFD, 0–1,000 μmol m^−2^ s^−1^) and FR fractions (0, 0.26, 0.45, and 0.63). FR treatments consistently increased *Φ*_PSII_, but when total photon flux density (TPFD, 400–800 nm) exceeded 1,000 μmol m^−2^ s^−1^, *Φ*_PSII_ rose abruptly. Nonfluorescent reference tests using white and black paper confirmed that FR induced spurious fluorescence signals, likely due to spectral overlap between FR photons and the PAM detection range (680–760 nm). Tilting the LED panel to reduce reflected FR eliminated the abrupt *Φ*_PSII_ peak but introduced unexpectedly increased *Φ*_PSII_ across treatments, likely due to probe-induced shading. These findings demonstrate that high-intensity FR can confound PAM-based CF measurements by producing spurious signals unrelated to plant physiology. Accurate and reliable assessment of photosynthetic performance under extended spectral lighting conditions requires careful management of lighting geometry and FR intensity.

## 1 Introduction

Light is one of the most critical environmental factors regulating photosynthesis and plant growth (Liu et al., 2024; Wu et al., 2025). Not only light intensity but also the spectral composition, or light quality, plays a vital role in determining plant morphology and physiology. In particular, chlorophyll absorbs light to drive the light reactions of photosynthesis, producing ATP and NADPH through the coordinated activity of photosystem II (PSII) and photosystem I (PSI) (Taiz and Zeiger, 2007; Lambers and Oliveira, 2019). Monitoring the efficiency of these processes, especially the quantum yield of PSII (*Φ*_PSII_), is a widely used approach to assess photosynthetic performance under various light conditions (Maxwell and Johnson, 2000; Baker, 2008; Murchie and Lawson, 2013; Swoczyna et al., 2022).

Far-red light (FR; 700–800 nm), traditionally excluded from the definition of photosynthetically active radiation (PAR; 400–700 nm), has received renewed attention for its distinct roles in plant growth and development. FR is well known to regulate shade avoidance, flowering, and stomatal behavior (Park et al., 2023). More recently, studies have demonstrated that FR can enhance photosynthetic performance by stimulating PSI activity, which indirectly improves PSII efficiency, as reflected in the *Φ*_PSII_ (Zhen and van Iersel, 2017; Murakami et al., 2018; Zhen et al., 2019). These findings have led to proposals for expanding the PAR concept to include FR, referred to as extended PAR (e-PAR) (Zhen et al., 2021).

**Figure 1:**
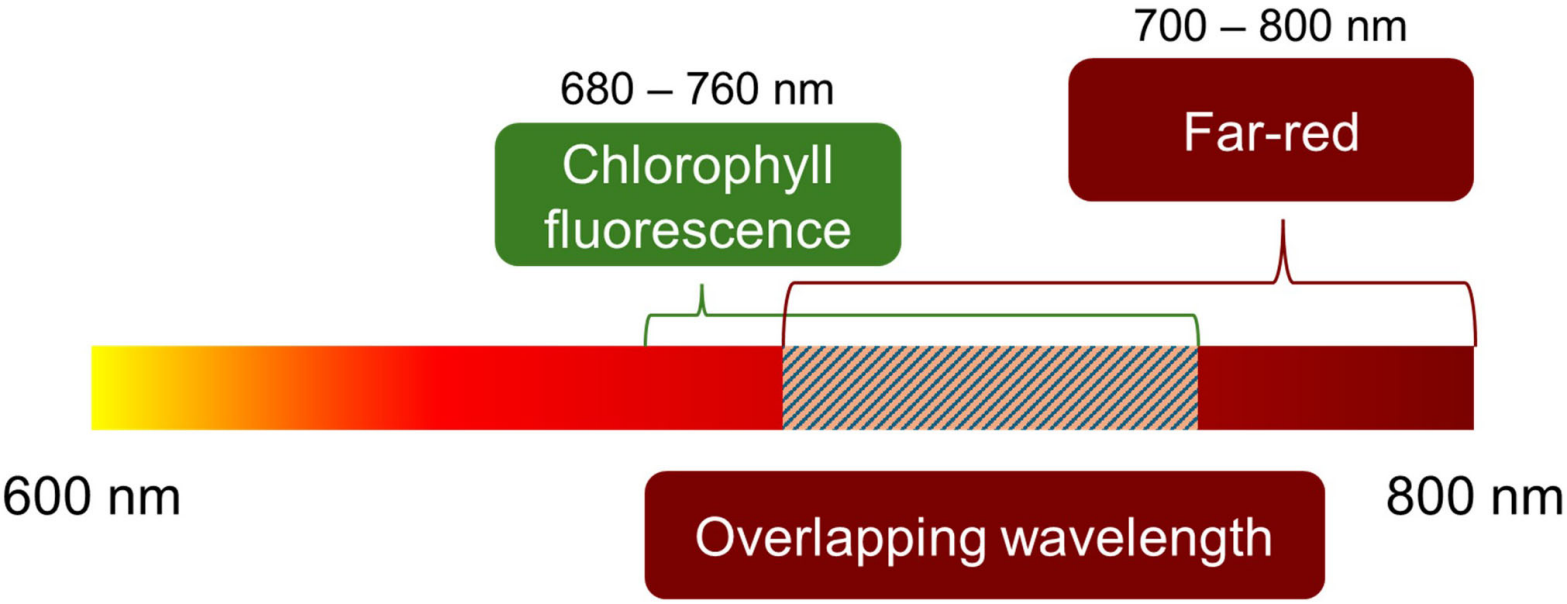
Spectral overlap between chlorophyll fluorescence (CF) emission and far-red (FR) irradiation.

**Figure 2:**
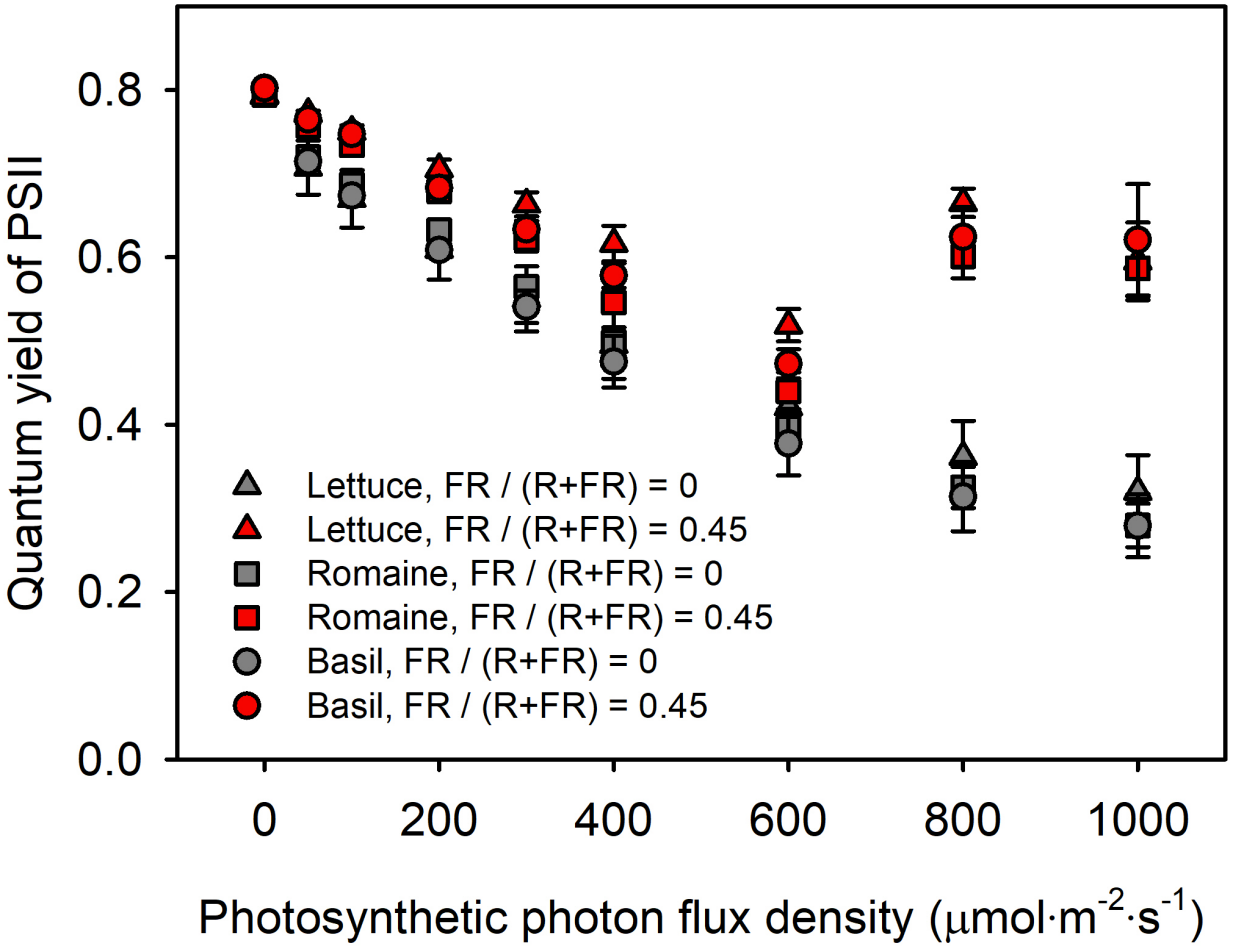
*Φ*_PSII_ of red leaf lettuce (triangles), romaine lettuce (squares), and sweet basil (circles) measured at PPFD levels from 0 to 1,000 μmol m^−2^ s^−1^. Grey and red symbols represent FR fraction of 0 and 0.45, respectively. Error bars indicate standard error (*n* = 3).

*Φ*_PSII_ is commonly assessed using pulse-amplitude modulation (PAM) fluorometers, which detect chlorophyll fluorescence (CF) emitted from leaves in the 680–760-nm range (Krause and Weis, 1991; Luedeker et al., 1997; Lambers and Oliveira, 2019; Legendre et al., 2021; Zhao et al., 2023). As mentioned above, FR has been highlighted in various studies and is increasingly incorporated into plant-lighting research. Since FR commonly overlaps with the detection range of PAM fluorometers (**Figure 1**), this overlap suggests that FR-induced misinterpretation may occur not only under controlled test conditions but also in many current research environments. Supporting this concern, in our preliminary tests, we observed anomalously high *Φ*_PSII_ values under strong light conditions (> 800 μmol m^−2^ s^−1^ PPFD) in both lettuce and sweet basil (**Figure 2**). These unexpected but consistent increases in the preliminary tests suggested measurement artifacts rather than genuine physiological responses.

We therefore hypothesized that FR light may interfere with PAM detection of CF, resulting in artificially inflated *Φ*_PSII_ estimates. The objective of this study was to quantify the extent of this interference and to identify the underlying mechanisms.

## 2 Materials and methods

### 2.1 Plant materials and growing conditions

Sweet basil (*Ocimum basilicum* L.) seeds (Asia Seed Co., Seoul, Korea) were sown in 128-cell plug trays filled with a germinating medium (Sunshine Mix No. 5; Sun-Gro Horticulture, Agawam, MA, USA) and grown in a greenhouse at Korea University, Seoul, Korea. Five weeks after sowing, seedlings were transplanted into 10-cm round plastic pots filled with a soilless medium (Sunshine Mix No. 4; Sun-Gro Horticulture) mixed with a controlled-release fertilizer (Multicote 6; NPK 14-14-14, Haifa Chemicals, Haifa, Israel) at 4 g·L^−1^. Transplanted seedlings were grown for 3 weeks in the greenhouse before being transferred to a customized plant growth chamber equipped with an LED dimming system and cultivated for an additional 5 weeks. The LED dimming system was equipped with six LED bars, each consisting of red (R; 600–700 nm; peak = 664 nm), green (G; 485–600 nm; peak = 524 nm), and blue (B; 400–485 nm; peak = 451 nm) LED chips (ESLEDs, Seoul, Korea). The average temperature and relative humidity of the greenhouse were 23.1°C ± 1.2°C and 32.6% ± 6.5%, respectively, and those of the growth chamber were 25.0°C ± 0.1°C and 65.8% ± 9.5% (mean ± SD). The average daily light integral in the greenhouse was 11.0 mol m^−2^ day^−1^ ± 4.0 mol m^−2^ day^−1^ (mean ± SD), and in the growth chamber, the canopy-level LED photosynthetic photon flux density (PPFD) was 200 μmol m^−2^ s^−1^ with a photoperiod of 16/8 h. The spectral composition of the LEDs was set to R:G:B = 44:18:38. The growing media in the pots were irrigated to saturation daily.

### 2.2 Light treatments for the light response curve

**Figure 3:**
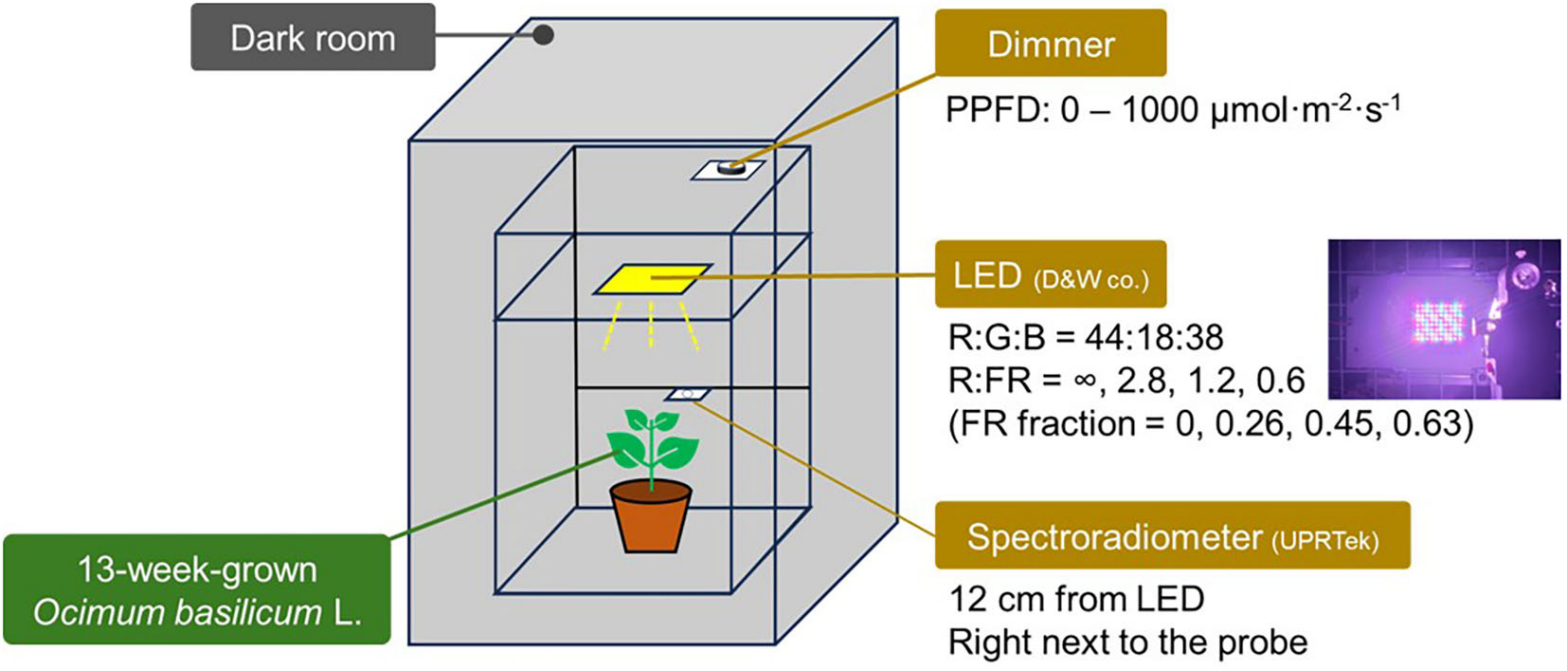
Schematic diagram of the experimental setup for examining far-red interference in chlorophyll fluorescence measurements. Sweet basil (*Ocimum basilicum* L.) was placed in a dark room, where a dimmable LED panel provided controlled light treatments. A spectroradiometer was positioned at canopy level to monitor spectral distribution and photon flux throughout the experiment.

Among the sweet basil plants grown in the growth chamber, three plants per treatment were randomly selected, and each plant was used for light response curve measurements (*n* = 3). Since the LED system in the chamber was designed for general plant cultivation and could not provide the high light intensities required for these measurements, we developed an independent LED setup specifically for light response curves. To construct the light response curves using a PAM fluorometer, we employed a customized LED dimming system comprising red (R, 600–700 nm, peak = 660 nm), green (G, 485–600 nm, peak = 525 nm), blue (B, 400–485 nm, peak = 450 nm), and FR (700–800 nm, peak = 730 nm) LED chips (D&W Co., Gwangmyeong, Korea) (**Figure 3**). The full width at half maximum (FWHM) of R, G, B, and FR are 16, 36, 18, and 46 nm, respectively. To measure the ratios and intensities of the R, G, B, and FR diodes at the uppermost, fully expanded leaf level, a spectroradiometer (PG200N, UPRtek, Miaodi County, Taiwan) was used to monitor light spectra. The spectroradiometer was positioned at the plant canopy level, 12 cm away from the LED panel, to adjust the diodes. The R:G:B ratio was 44:18:38, with PPFD levels adjusted sequentially to 0, 50, 100, 200, 300, 400, 600, 800, and 1,000 μmol m^−2^ s^−1^ to construct light response curves. FR treatments were imposed at four FR fractions (0, 0.26, 0.45, 0.63) across all PPFD levels. The FR fraction was determined with R and FR photon flux density (PFD) as FR PFD/(R PFD + FR PFD).

### 2.3 Chlorophyll fluorescence parameters

All fluorescence measurements were conducted in a darkroom to minimize external light interference, using a PAM fluorometer (Mini-PAM-II; Heinz Walz GmbH, Effeltrich, Germany). The maximum quantum yield of PSII (*F_v_*/*F_m_*) was determined after 20 min of dark adaptation by measuring the minimal fluorescence level (*F_o_*) in the dark and the maximal fluorescence level (*F_m_*) using a saturating light pulse. *F_v_*/*F_m_* was calculated as *F_v_*/*F_m_* = (*F_m_* − *F_o_*)/*F_m_*. Under actinic light, the steady-state fluorescence level (*F_t_*) and the maximal fluorescence level (*F’_m_*), measured using a saturating light pulse applied to light-adapted leaves, were used to calculate *Φ*_PSII_ as (*F’_m_* − *F_t_*)/*F’_m_* (Maxwell and Johnson, 2000; Murchie and Lawson, 2013). After dark adaptation, leaves were preacclimated for 20 min at 50 μmol m^−2^ s^−1^, followed by 7 min of acclimation at each subsequent PPFD (100–1,000 μmol m^−2^ s^−1^). *Φ*_PSII_ was recorded every 30 s, five times per light level. *F_o_*, measured in complete darkness, converged to zero.

**Figure 4:**
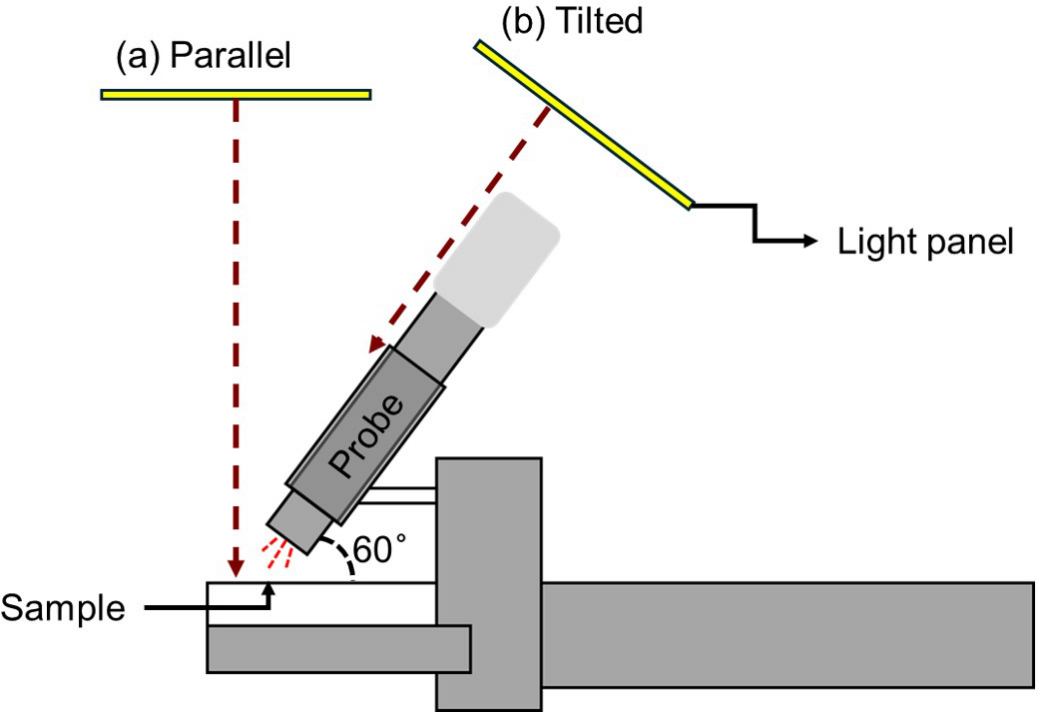
Schematic illustration of far-red (FR) light interference in PAM fluorescence measurements. **(a)** In the parallel LED panel configuration, reflected FR photons entered the probe and were misinterpreted as chlorophyll fluorescence. **(b)** Tilting the LED panel minimized direct reflection into the probe, reducing FR-induced artifacts. The PAM probe was positioned at a 60° angle relative to the sample surface. Red dashed arrows indicate the trajectory of FR light.

To test potential FR interference, *F_t_* and *F’_m_* from black or white paper were also measured under the four FR fractions as nonfluorescent reference tests. In addition, two panel orientations were compared: (a) parallel to the leaf surface (perpendicular light direction to the surface) and (b) perpendicular to the PAM probe (aligning the light direction with the probe by tilting the panel to a 60° angle) (**Figure 4**). For these tests, light response curves were obtained at FR fractions of 0 and 0.45, following the same sequence as described above.

### 2.4 Statistical analysis

Differences in parameter means were analyzed by a nonparametric Kruskal–Wallis test for comparisons between two FR fractions (0 and 0.45) or between nontilted and tilted panel treatments. Comparisons among four FR fractions (0, 0.26, 0.45, and 0.63) were analyzed by one-way ANOVA followed by Tukey’s honestly significant difference at *α* = 0.05. All treatments included three biological replicates (*n* = 3). Statistical analyses were performed using SAS 9.4 (SAS Institute, Cary, NC, USA).

## 3 Results

**Figure 5:**
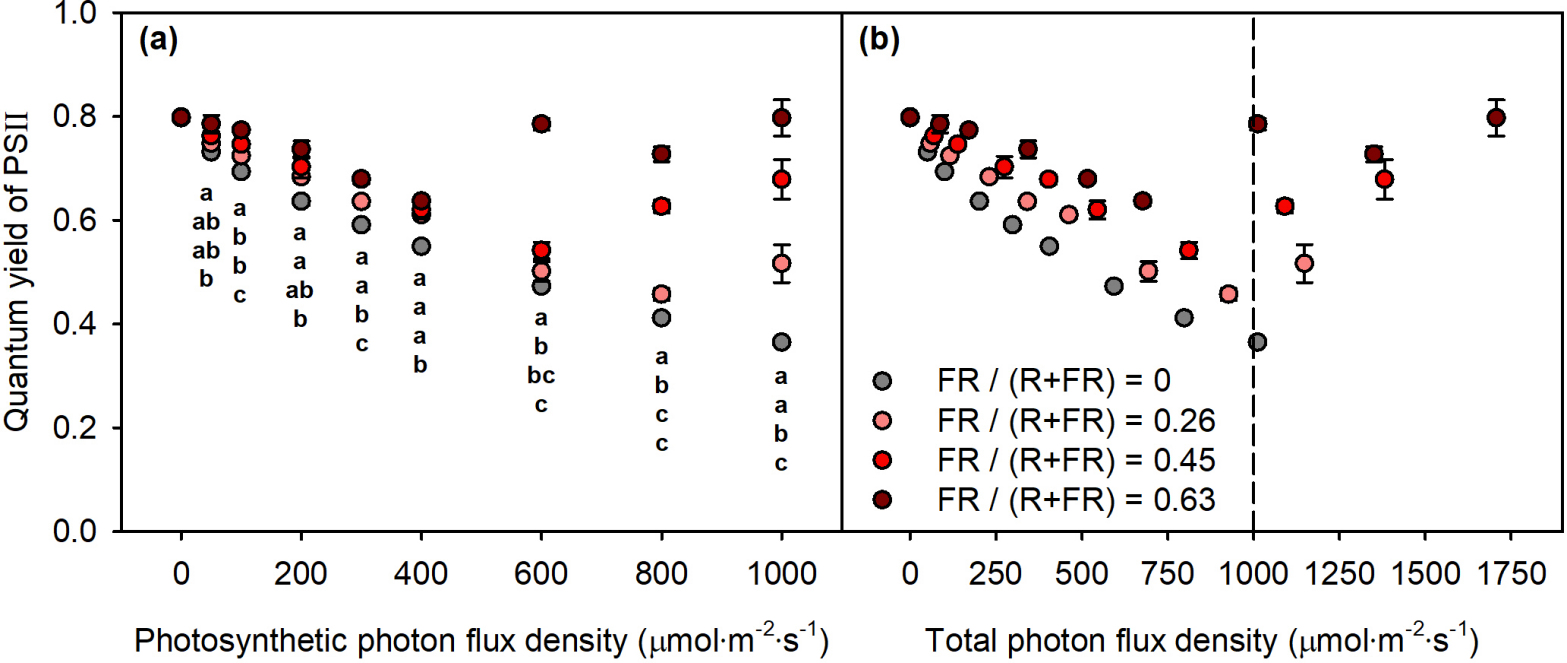
*Φ*_PSII_ of sweet basil at different **(a)** PPFD and **(b)** TPFD levels. Grey, light red, red, and dark red symbols indicate FR fractions of 0, 0.26, 0.45, and 0.63, respectively. Means followed by the same letter within the same PPFD are not significantly different following Tukey’s honestly significant difference at *α* = 0.05. Error bars indicate standard error (*n* = 3).

Regardless of FR fraction, *Φ*_PSII_ declined with increasing PPFD. However, *Φ*_PSII_ of sweet basil was consistently higher under FR treatments than under non-FR treatments across all PPFD levels (**Figure 5**). At PPFD levels of 50–400 μmol m^−2^ s^−1^, FR treatments increased *Φ*_PSII_ up to 17% relative to the non-FR treatment. However, when PPFD exceeded 600 μmol m^−2^ s^−1^, FR treatments increased *Φ*_PSII_ up to 121% relative to the non-FR treatment. Since PPFD does not account for the contribution of FR photons, we reanalyzed the data using total photon flux density (TPFD; 400–800 nm) to better represent the actual light environment. On a TPFD basis, FR treatments produced an abrupt increase in *Φ*_PSII_ once TPFD exceeded 1,000 μmol m^−2^ s^−1^, compared to the non-FR treatment (**Figure 5b**).

**Figure 6:**
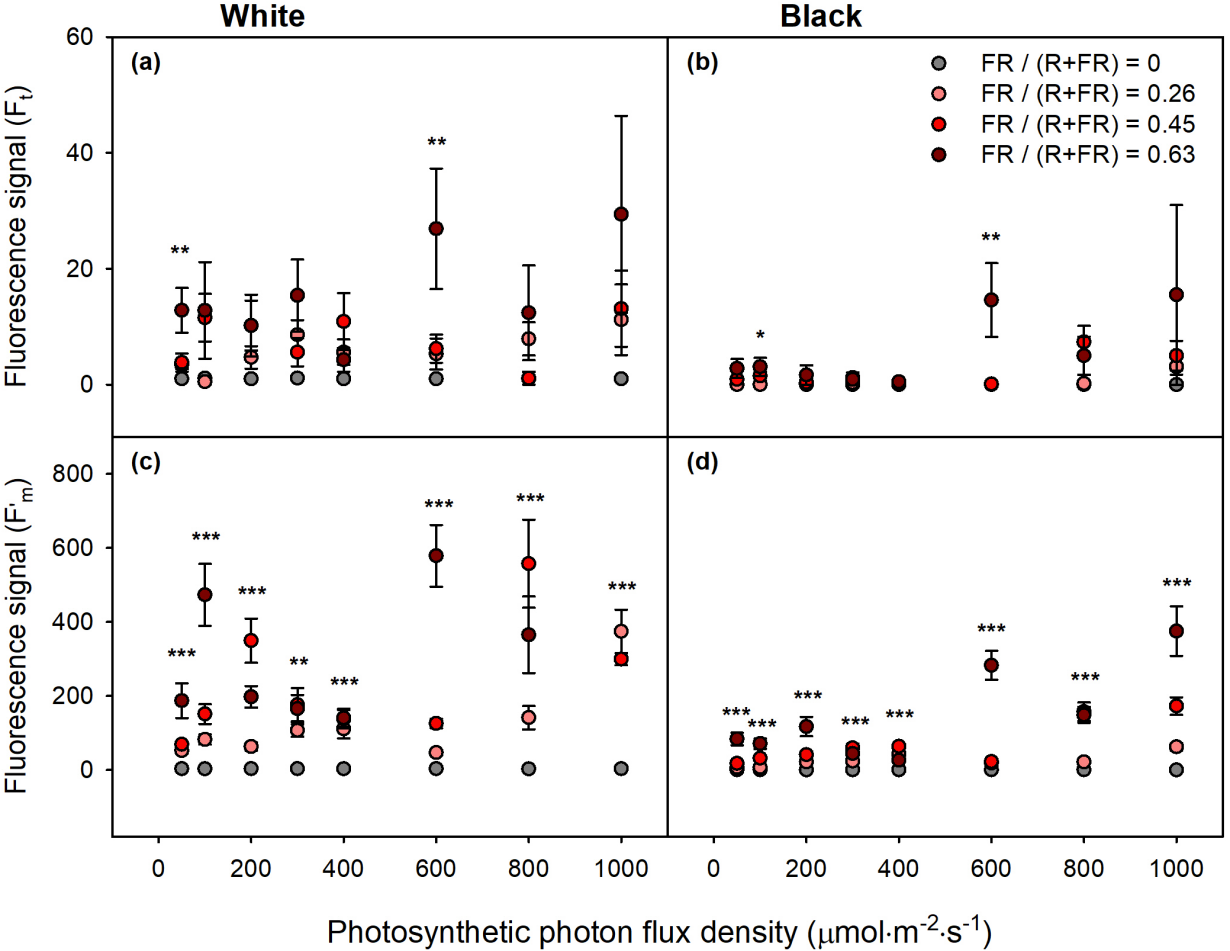
**(a, b)***F_t_* and **(c, d)***F’_m_* measured from white paper **(a, c)** and black paper **(b**, **d)**. Grey, light red, red, and dark red symbols indicate FR fractions of 0, 0.26, 0.45, and 0.63, respectively. ^*^p < 0.05, ^**^p < 0.01, and ^***^p < 0.001 indicate statistically significant differences at each PPFD level. Error bars indicate standard error (*n* = 3).

To test whether these responses were caused by direct reflection of FR to the probe, *F_t_* and *F’_m_* were measured at each PPFD level using both white and black papers. Under the non-FR treatments, *F_t_* and *F’_m_* remained close to zero. In contrast, under FR treatments, the signals increased to levels detectable by the fluorometer, and these fluctuations were consistently observed at higher FR fractions (**Figure 6**). This indicates that reflected FR photons were detected as CF.

**Figure 7:**
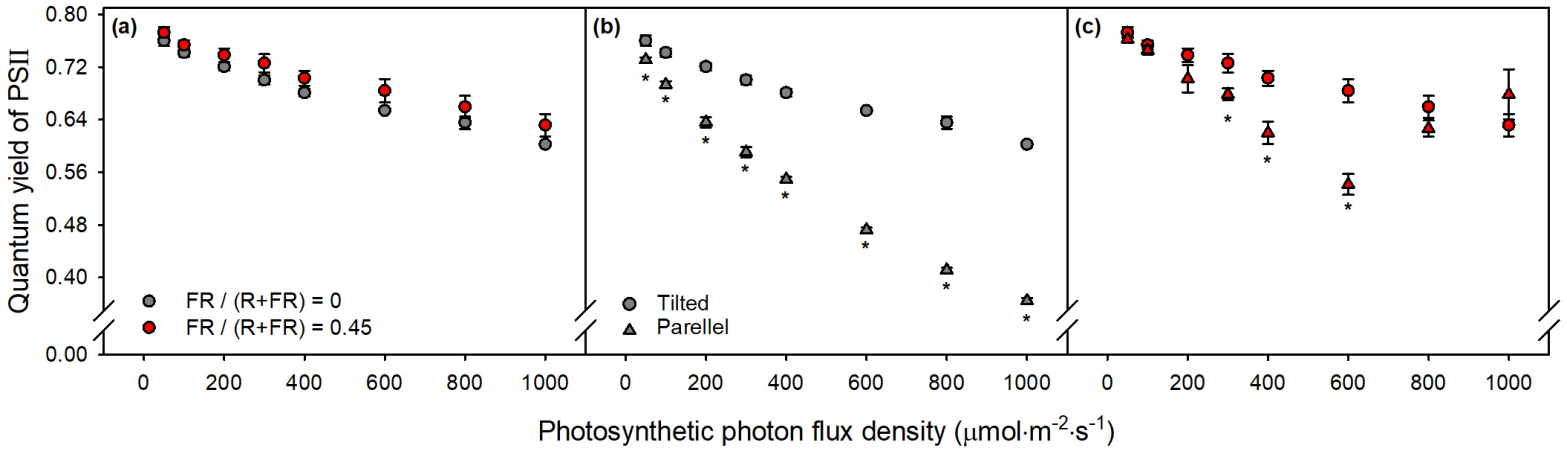
**(a)***Φ*_PSII_ of sweet basil measured with a tilted LED panel (perpendicular to the PAM probe). **(b)***Φ*_PSII_ of non-tilted LED panel (triangles) and tilted LED panel (circles) under FR fraction of 0. **(c)***Φ*_PSII_ of non-tilted LED panel (triangles) and tilted LED panel (circles) under FR fraction of 0.45. Grey and red symbols indicate FR fractions of 0 and 0.45, respectively. ^*^p < 0.05 indicate statistically significant difference at each PPFD level. Error bars indicate standard error (*n* = 3).

When the LED panel was tilted perpendicular to the PAM probe, the anomalous FR-induced spike disappeared (**Figure 7**). Unlike the parallel panel configuration (**Figure 5a**), the tilted setup eliminated the abrupt FR-induced increase in *Φ*_PSII_. No statistically significant differences in *Φ*_PSII_ were observed between the FR and non-FR treatments across all PPFD levels (**Figure 7a**). Even without FR, the tilted panel treatment produced higher *Φ*_PSII_ (**Figure 7b**). Under FR treatments, the tilted panel treatment appeared to yield higher *Φ*_PSII_ at 300, 400, and 600 μmol m^−2^ s^−1^, but no differences were observed at other PPFD levels (**Figure 7c**).

## 4 Discussion

Under moderate light conditions (TPFD < 1,000 μmol m^−2^ s^−1^), supplemental FR light increased *Φ*_PSII_, consistent with previous studies showing that FR enhances PSI excitation and facilitates plastoquinone A oxidation (Baker and Rosenqvist, 2004; Baker, 2008; Zhen and van Iersel, 2017). However, at higher TPFD levels (> 1,000 μmol m^−2^ s^−1^), the abrupt rise in *Φ*_PSII_ contradicts the established understanding that *Φ*_PSII_ generally declines as light intensity increases, due to increased photochemical and nonphotochemical quenching (Maxwell and Johnson, 2000; Murchie and Lawson, 2013). The magnitude and abruptness of this increase suggest measurement artifacts, rather than genuine physiological responses, may be responsible. It was also observed that, with more intense FR application, the increase in *Φ*_PSII_ tended to occur at lower PPFD. As FR is known to generate fluctuating artifacts, the likelihood of abnormal *Φ*_PSII_ increased as the FR fraction increased. As PPFD rose, FR intensity also increased, and the rate of FR increase became steeper with higher FR fractions, resulting in a greater chance of abnormal *Φ*_PSII_ appearing at lower PPFD. However, further studies are needed to clarify why the *Φ*_PSII_ artifacts occurred at TPFD levels above 1,000 μmol m^−2^ s^−1^.

Signal saturation occurred at high FR intensity on both white and black papers in the absence of plants, indicating that the PAM fluorometer detected reflected FR photons as CF. This effect was particularly pronounced on white paper, likely due to its high reflectivity, whereas black paper exhibited reduced but still detectable fluctuations. In contrast, *F_t_* and *F’_m_* under non-FR treatment remained at zero regardless of PPFD levels. Therefore, this interference likely arose from FR directly reflected to the probe and from the spectral overlap between FR (700–800 nm) and the CF emission range (680–760 nm) (Krause and Weis, 1991; Lambers and Oliveira, 2019). Since the probe was angled at approximately 60° relative to the sample surface, reflected FR could enter the detector directly, resulting in inflated *Φ*_PSII_ values.

Tilted configuration minimized direct reflection into the detector, eliminating the spurious increase in *Φ*_PSII_. These results indicate that accurate PAM measurements under FR illumination require careful control of light geometry to prevent reflected FR from being misinterpreted as CF. However, tilting also introduced new artifacts through probe shading, which reduced effective excitation and artificially increased *Φ*_PSII_. Thus, while panel tilting effectively reduces FR interference, it introduces a separate distortion that must be carefully considered in experimental design. Researchers using a PAM fluorometer under FR-rich conditions should account for optical geometry, and complementary methods may be necessary to confirm true physiological responses. Such approaches may include using FR-blocking filters at the probe entrance to exclude interference from photons, or an integrating sphere-based fluorometer setup that provides diffuse and uniform illumination to minimize artifacts by direct reflection.

## 5 Conclusions

Our findings demonstrate that high-intensity FR light can interfere with PAM-based chlorophyll fluorescence measurements by generating artifactual signals within the CF detection range. Tilting the light source reduced FR-induced interference but introduced shading artifacts, underscoring the combined influence of spectral and geometric factors. Accurate CF assessment under extended spectral conditions requires rigorous control of light geometry and validation beyond PAM fluorometry, particularly as the definition of PAR expands to include FR.

## Data Availability

The original contributions presented in the study are included in the article/supplementary material. Further inquiries can be directed to the corresponding author.
